# NLRP3-dependent pyroptosis exacerbates coxsackievirus A16 and coxsackievirus A10-induced inflammatory response and viral replication in SH-SY5Y cells

**DOI:** 10.1016/j.virusres.2024.199386

**Published:** 2024-05-06

**Authors:** Yajie Hu, Wei Zhao, Yaming Lv, Hui Li, Jiang Li, Mingmei Zhong, Dandan Pu, Fuping Jian, Jie Song, Yunhui Zhang

**Affiliations:** a Department of Respiratory Medicine, The First People's Hospital of Yunnan Province, China; b The Affiliated Hospital of Kunming University of Science and Technology, Kunming, Yunnan, China; c National and Local Engineering Center for Infectious Biological Products, Institute of Medical Biology, Chinese Academy of Medical Science and Peking Union Medical College, Kunming, China

**Keywords:** Coxsackievirus A16 (CV-A16), Coxsackievirus A10 infection, Inflammatory response, Viral replication, NLRP3-inflammasome-mediated pyroptosis, SH-SY5Y cells

## Abstract

Coxsackievirus A16 (CV-A16) and coxsackievirus A10 (CV-A10), more commonly etiological agents of hand, foot and mouth disease (HFMD), are capable of causing severe neurological syndromes with high fatalities, but their neuropathogenesis has rarely been studied. Mounting evidence indicated that pyroptosis is an inflammatory form of cell death that might be widely involved in the pathogenic mechanisms of neurotropic viruses. Our study was designed to examine the effects of NLRP3-mediated pyroptosis in CV-A16- and CV-A10-induced inflammatory neuropathologic formation. In this work, it was showed that SH-SY5Y cells were susceptible to CV-A16 and CV-A10, and meanwhile their infections could result in a decreasing cell viability and an increasing LDH release as well as Caspase1 activation. Moreover, CV-A16 and CV-A10 infections triggered NLRP3-mediated pyroptosis and promoted the release of inflammatory cytokines. Additionally, activated NLRP3 accelerated the pyroptosis formation and aggravated the inflammatory response, but inhibited NLRP3 had a dampening effect on the above situation. Finally, it was further revealed that NLRP3 agonist enhanced the viral replication, but NLRP3 inhibitor suppressed the viral replication, suggesting that NLRP3-driven pyroptosis might support CV-A16 and CV-A10 production in SH-SY5Y cells. Together, our findings demonstrated a mechanism by which CV-A16 and CV-A10 induce inflammatory responses by evoking NLRP3 inflammasome-regulated pyroptosis, which in turn further stimulated the viral replication, providing novel insights into the pathogenesis of CV-A16 and CV-A10 infections.

## Highlights

• The study highlighted that CV-A16 and CV-A10 infections triggered cell death probably involved in NLRP3-mediated pyroptosis and inflammatory response.
• The study demonstrated that NLRP3 may support CV-A16 and CV-A10 replication in SH-SY5Y cells.
• The study confirmed that NLRP3-dependent pyroptosis might be an important contributing factor for inducing exacerbated inflammation during CV-A16 and CV-A10 infections.

## 1 Introduction

Hand, foot, and mouth disease (HFMD) is a common pediatric infectious disease, frequently affecting children below the age of five, which is characterized by typical clinical manifestations, such as low-grade fever, sore throat, poor appetite, vesicular rashes on hands, feet and buttocks, and ulcers in the oral mucosa (Aswathyraj et al., 2016). Under normal conditions, the symptoms of HFMD spontaneously resolved in a few days, but sometimes there were a small number of patients experiencing severe neurological complications, including meningitis, aseptic encephalitis, acute flaccid paralysis, and even fatal pneumonia and acute viral myocarditis (Nayak et al., 2022). Currently, multiple, highly contagious human enteroviruses species A (HEV-A) types, especially enterovirus A71 (EV-A71) and coxsackievirus A16 (CV-A16) have been identified to be the major etiological agents of HFMD (Esposito and Principi, 2018; Zhu et al., 2023). However, with the availability of the inactivated EV-A71 vaccine, the infection rate of EV-A71 was significantly reduced and the pathogenetic spectrum of HFMD has also changed (Liang and Wang, 2014; Mao et al., 2016). CV-A16, CV-A10 and CV-A6 have gradually emerged to be predominant in HFMD outbreaks across the world (Bian et al., 2019; Kimmis et al., 2018; Mao et al., 2014). Historially, previous studies have found that patients infected with EV-A71 often tend to occur serious central nervous system (CNS) complications, while patients infected with CV-A16 and CV-A10 usually show mild, self-limited viral syndrome. In recent years, growing epidemiological surveys have indicated that neurologic and cardiopulmonary involvement were also observed in some severe and fatal cases of CV-A16 and CV-A10 infections (Chen et al., 2021; Gonzalez et al., 2019; Wang et al., 2004a). For example, aseptic meningitis, encephalitis, acute flaccid paralysis, pneumonitis, pulmonary edema, pulmonary hemorrhage, or myocarditis were reported in some CV-A16 infections (Goto et al., 2009; Tao et al., 2014; Wang et al., 2004a). An Indian study of enterovirus molecular identification in patients with aseptic meningitis found seven enterovirus serotypes, including CV-A10, which confirmed that CV-A10 could induce CNS complications in patients (Kumar et al., 2013). Meanwhile, B.S. Astrup has reported a case of sudden unexplained death in an infant caused by CV-A16 infection (Astrup et al., 2016) and Meghan E. Fuschino has also detected CV-A16 in multiple tissues of a fatal infant sepsis case (Fuschino et al., 2012). Moreover, mounting evidence has uncovered that CV-A16 or CV-A10 or their co-infection with other enteroviruses could cause more serious outcomes as compared to EV-A71 infection, which have been underestimated (Kimmis et al., 2018; Mao et al., 2014). Unfortunately, effective antiviral agents and vaccines for CV-A16 and CV-A10 related HFMD is unavailable yet (Zhang et al., 2022a). Thus, investigations into the underlying molecular mechanisms of CV-A16 and CV-A10 infections are very important and necessary, which can provide a basis for the design of future therapies against CV-A16 and CV-A10 infections.

Viruses, a special class of organisms, are strictly parasitic on cells and effectively utilize the infected cell transcription and translation machinery to complete their replication life cycle (Park et al., 2023; Simmonds and Aiewsakun, 2018). Cell death is a critical, unavoidable antiviral response to defense against virus invasion, which deprives the survival environment of viruses (Imre, 2020; Tummers and Green, 2022). However, a large number of studies have also confirmed that cell death cannot only curb virus replication, but also enhance virus dissemination, further contributing to tissue injury and worsening of viral diseases (Imre, 2020; Newton et al., 2024). Presently, three major ways of regulated cell death mechanisms are observed following virus infection, namely, apoptosis, necroptosis, and pyroptosis, which plays a fundamental role in infectious disease pathogenesi (D'Arcy, 2019). Apoptosis, the most extensively studied cell death mechanism, is a non-lytic form of cell death which is marked by the formation of apoptotic bodies after the intracellular contents from dying cells disintegrate and are sequestered into small fragments, while necroptosis and pyroptosis both are the lytic form of cell death which results in the breakdown of membrane integrity and the release of intracellular substances into extracellular space (Ketelut-Carneiro and Fitzgerald, 2022). Nonetheless, the release of intracellular substances, including damage-associated molecular patterns, inflammtory cytokines, etc., is a critical step that directly leads to the activation of the inflammatory response (Bertheloot et al., 2021). Pyroptosis is normally initiated through the canonical pathway mediated by caspase 1 and the noncanonical pathway mediated by caspase-4, caspase-5 or caspase-11, and the canonical pathway requires the formation of inflammasome, a cytosolic protein complex consisting of one of several sensor proteins, such as Nod-like receptor family pyrin domain containing 3 (NLRP3), absent in melanoma 2 (AIM2), IFN-gamma-inducible protein-16 (IFI16) and so on (Bertheloot et al., 2021; Yu et al., 2021). Based on its ability to induce the body's inflammatory response, pyroptosis exhibits a significant role in viral infections (Burdette et al., 2021; Liu et al., 2021). For example, respiratory syncytial virus (RSV) has been shown to activate the NLRP3 inflammasome and then result in secretion of IL-1β, which finally contributes to the pathology associated with RSV infection (Shen et al., 2018). The pyroptosis pathway was activated by pseudorabies virus (PRV) infection via elevating the expression levels of NLRP3, Caspase1, Gasdermin-D and IL-1β/18, which one of the main reasons of the rapid death of mice infected with PRV (Ye et al., 2021). Influenza A virus (IAV) infection commits the cell to cell death in the form of pyroptosis which persistently activates and releases the pro-inflammatory cytokines IL-1β and IL-18, ultimately exacerbating inflammation and leading to respiratory failure (Wan et al., 2022). Actually, it has been reported that many human enteroviruses could cause cellular pyroptosis (Wang et al., 2022). For instance, CVB3 infection dramatically increased the expression of caspase‐1, NLRP3, IL‐18, and IL‐1β, and the use of a caspase‐1 inhibitor could significantly inhibit viral replication and reduce myocarditis, which indicated that CVB3 infection is closely related to pyroptosis (Zhang et al., 2022b). EV-A71 could induce NLRP3 or AIM2 mediated pyroptosis which in turn exhibits a role in limiting EV‐A71 replication and eventually influences the progression of EV-A71 infection (Guo et al., 2023; Yogarajah et al., 2017). Furthermore, our previous study has also verified that EV-A71 initiated inflammatory pryoptosis via activating the hsa_circ_0045431/ hsa_miR_584/NLRP3 regulatory axis (Hu et al., 2023). However, the detailed roles of pyroptosis in CV-A16 and CV-A10 infections are still unclear. Hence, the study aims to explore the potential mechanism of pyroptosis in the process of CV-A16 and CV-A10 infections.

## 2 Materials and methods

### 2.1 Cell culture and treatment

The human neuroblastoma cell line SH-SY5Y were purchased from BeNa Culture Collection (Beijing, China) and cultured in Dulbecco's Modified Eagle Medium (DMEM) (Solarbio, Beijing, China) containing 10 % fetal bovine serum (FBS; Corning, USA) and 1 % penicillin-streptomycin solution (Procell, China) at 37 °C and 5 % CO_2_ under saturated humidity.

CV-A16 (subgenotype B, GenBank NO. JN590244.1), isolated from an HFMD patient in Guangxi, China, in 2010, and CV-A10 (subgenotype C, GenBank NO. MN557275), isolated during an epidemic in Xiangyang, China, in 2017, were used in this study. For CV-A16 or CV-A10 infections, cells were infected at an MOI of 0.01, 0.1 or 1, respectively. After 2 h of incubation with CV-A16 or CV-A10, cells were supplemented with 10 % FBS and then incubated for the indicated time. Moreover, to promote or inhibit NLRP3 activation, cells were pre-treated with Nigericin sodium salt or MCC950 sodium for 30 min, respectively. Then, cells were incubated with CV-A16 or CV-A10 in the presence of Nigericin sodium salt or MCC950 sodium for 2 h, washed with PBS, and finally cultured with DMEM containing Nigericin sodium salt or MCC950 sodium. Nigericin sodium salt and MCC950 sodium were both purchased from MCE, a leading global supplier of research chemicals and bioactive compounds. According to the concentration recommendation of *in vitro* cell experiment in the manual and some literature consulted (He et al., 2022; Liu et al., 2019), we chose the concentration of 10 μM for this experiment.

### 2.2 Immunofluorescence (IF) assay

IF staining was performed using treated SH-SY5Y cells on poly-lysine‐coated glass coverslips. Cells were washed twice with pre-cooled PBS and then fixed with 4 % paraformaldehyde for 30 min. Afterwards, cells were permeabilized with 0.2 % Triton X-100 in PBS at room temperature for 5 min, and washed twice with PBS. Non-specific binding sites were blocked by 0.5 % bovine serum albumin (BSA) for 30 min. After three further washes with PBS, cells were incubated overnight at 4 °C with anti-VP1 (For CV-A16, 1:1000 dilution; Millipore, USA) or anti-VP1 (For CV-A10, 1:1000 dilution; GeneTex, China) and anti-Caspase1 (1:100 dilution; Affinity, UAS), followed by fluorescein isothiocyanate (FITC)-conjugated donkey anti-mouse IgG and Alexa Fluor 594-conjugated donkey anti-rabbit IgG secondary antibodies (1:300 dilution; CST, USA), for 2 h at room temperature. After three final washes, the nuclei were counterstained with diamidino-2-phenylindole (DAPI; 1:1000 dilution; Beyotime, China) for 5 min, and then washed three more times with PBS. Finally, the cells were analyzed using a confocal laser scanning microscope (Leica, Germany).

### 2.3 Quantitative real-time polymerase chain reaction (qRT-PCR)

The total RNA was extracted from cells by virtue of the TRIzol reagent (Invitrogen, USA) following the manufacturer's protocol. A NanoDrop 2000c (Thermo Scientific, USA) instrument was used to assess RNA quality. The complementary DNA (cDNA) was carried out starting from 500 ng of total RNA. Then, qRT‐PCR was executed using the SYBR Master Mix (ABI, USA) on an ABI 7500 system (ABI, USA). The primer sequences for the examined genes were listed in Table 1. The glyceraldehyde-3-phosphatedehydrogenase (GAPDH) was used as an internal references and all the relative gene expression levels were calculated using the 2^−ΔΔCt^ method.

**Table 1:** Primer sequences table.

Gene	Primer sequences
NLRP3	Forward: 5′-ATCTTTGCTGCGATCAACAGGCG-3′ Reverse: 5′-CGCGTTCCTGTCCTTGATAGAGT-3′
ASC	Forward: 5′-AGACATGGGCTTACAGGA-3′ Reverse: 5′-CTCCCTCATCTTGTCTTGG-3′
Caspase1	Forward: 5′-GCCTGTTCCTGTGATGTGGA-3′ Reverse: 5′-CTTCACTTCCTGCCCACAGA-3′
Gasdermin D	Forward: 5′-GCAGCCTGAGCACAAAGTCCT-3′ Reverse: 5′-CCTCCACCTCCTTCTGCGTCT-3′
IL-1β	Forward: 5′-AGCTCGCCAGTGAAATGATGG-3′ Reverse: 5′-TAGTGGTGGTCGGAGATTCG-3′
IL-18	Forward: 5′-CGCTTCCTCTCGCAACAAAC-3′ Reverse: 5′-ATTCCAGGTTTTCATCATCTTCAGC-3′
GAPDH	Forward: 5′-ACAACTTTGGTATCGTGGAAGG-3′ Reverse: 5′-GCCATCACGCCACAGTTTC-3′

### 2.4 Virus stocks examination

Virus stocks were titrated by standard median cell culture infective doses (CCID50) assay on Vero cells. The supernatants of SH-SY5Y cells were collected and centrifuged, and 10-fold diluted. 100 μl of supernatants and virus stock were added into Vero cells which were seeded in 96-well plate at 90 % confluence, at 37 °C for about 72 h. Cytopathic effect of each of well was counted after cells were fixed with 4 % formaldehyde and stained by 0.5 % crystal violet. In the end, CCID50/1 ml values were calculated by the Reed-Muench method.

### 2.5 Cell proliferation assay

The cells were seeded into 96 well sets at a density of 2 × 10^4^ cells/well and after reaching 80–90 % confluence, cells were treated as the above mentioned. Cell viability was assessed at 0, 6, 12, 24, 48 and 72 h using the Cell Counting Kit (CCK)‐8 (Dojindo Molecular Technologies, Japan). A microplate reader (Tecan, Switzerland) was used to test the optical absorbance values at 450 nm.

### 2.6 Lactate dehydrogenase (LDH) cytotoxicity assay

The supernatants were harvested and cell death was measured by a LDH Cytotoxicity Assay Kit (Beyotime, China) according to the instructions provided by the company. Briefly, 120 μl of cell supernatant was collected and centrifuged at 400 × g for 5 min. Then, 60 μL of LDH test working fluid was added to the samples and incubated at room temperature for 30 min. The absorbance was measured at 490 nm in a spectrophotometric microplate reader.

### 2.7 Activated caspase1 measurement

SH-SY5Y cells were plated overnight and treated as described above. At the indicated time, the supernatants were collected for caspase1 activity measurement using a Caspase1 Activity Assay Kit (Beyotime, China) according to the manufacturer's instructions.

### 2.8 Flow cytometry for cell death assessment

Cells (1 × 10^5^ per well) were seeded into 24-well plates and cultured with CV-A16 or CV-A10 infection. Cells at 0, 6, 12, 24, 48 and 72 h were then resuspended in binding buffer and stained in the dark with 5 μL Annexin V-fluorescein isothiocyanate (FITC) and propidium iodide (PI; Solarbio, China) for 10 min. The different kind of death cells were assessed through a NovoCyte flow cytometer (ACEA Bioscience, USA).

### 2.9 Western blot

The protein was prepared on ice using a total protein extraction kit (Solarbio, China) and protein concentration was determined by a BCA protein assay Kit (BioRad, USA), followed by denaturation at 98 °C for 10 min. Equal amounts of proteins were subsequently subjected to 10 % sodium dodecyl sulfate-polyacrylamide gel electrophoresis (SDS-PAGE) and transferred onto polyvinylidene difluoride (PVDF) membranes (Millipore, USA). After the blockage of nonspecific binding sites with 5 % skim milk at room temperature for 2 h, the PVDF membranes were incubated with primary antibodies against NLRP3 (1:1000 dilution; Affinity, UAS), ASC (1:2000 dilution; Affinity, UAS), Caspase 1 (1:1500 dilution; Affinity, UAS), Gasdermin D (1:1000 dilution; Affinity, UAS), IL-1β (1:800 dilution; Affinity, UAS), IL-18 (1:800 dilution; Affinity, UAS), VP1 (1:1000 dilution; China) or anti-GAPDH (1:5000 dilution; CST, USA) at 4 °C overnight. After TBST washing 3 times, the membranes were incubated with secondary antibodies (1:2000; CST, USA) for 1 h at room temperature. Finally, the protein bands were visualized via enhanced chemiluminescence reagent (Beyotime, China). The gray values were analyzed with GAPDH as a loading control by QuantityOne software (Bio-Rad, USA).

### 2.10 Enzyme-linked immunosorbent assay (ELISA)

The concentration of culture supernatants about IL-1β and IL-8 were measured by commercial ELISA kits (Neobioscience, China) according to manufacturer's instructions. In brief, the supernatants were collected and incubated with reaction solution and subsequently with stop solution. Captured cytokines were detected by reading absorbance values in a microplate reader (Tecan, China). Analysis was done using ESACalc software.

### 2.11 Cytokine quantification analysis with flow cytometry

The supernatants of the cultured cells were collected for inflammatory cytokines quantification. The protein levels of TNF-α, IL-12, IL-4, IL-17, IL-8, IFN-γ, IL-10, IL-1β, IL-6, IL-2, IFN-α and IL-5 were measured with a Bio-Plex cytokine assay (RAISECARE, China) following a standard procedure provide by the merchant's instructions. Flow cytometric analysis was performed on a LEGENDplex v8.0 software.

### 2.12 Statistical analysis

All statistical analyses were performed using GraphPad Prism 7.0 software. The data were presented as means ± standard error of the mean (SEM). The results were estimated by Student's *t*-test or one-way analysis of variance (ANOVA) with Tukey's post-hoc test. *P* < 0.05 was considered statistically significant.

## 3 Results

### 3.1 CV-A16 and CV-A10 both infects the SH-SY5Y cells

**Fig. 1:**
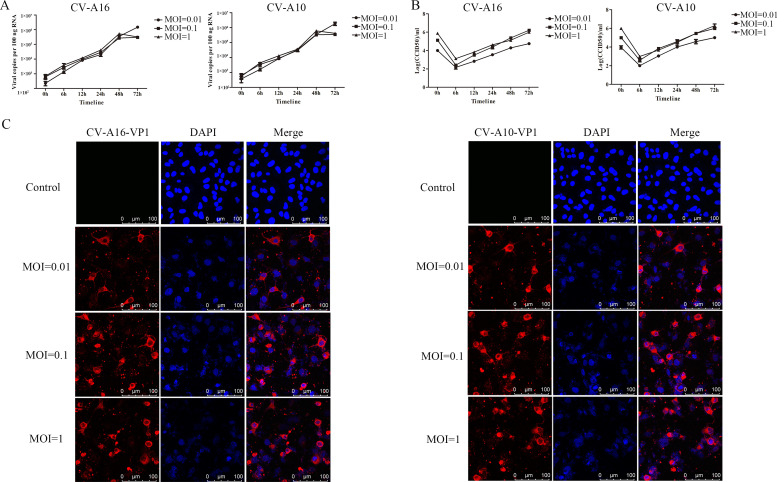
Proliferation of CV-A16 and CV-A10 in SH-SY5Y cells inoculated with different MOIs. (A) Viral loads was detected by qRT-PCR in CV-A16- and CV-A10-infected SH-SY5Y cells at MOIs of 0.01, 0.1 and 1. (B) Virus titer was examined by CCID_50_ method using SH-SY5Y infected samples of CV-A16 and CV-A10 on Vero cells. (C) Viral infection was assessed with IF staining in CV-A16- and CV-A10-infected SH-SY5Y cells with MOIs of 0.01, 0.1 and 1 at 72 h.

To explore if CV-A16 or CV-A10 could infect SH-SY5Y cells and replicate in SH-SY5Y cells, the viral loads, virus titer and virus location were examined with MOI=0.01, 0.1 and 1 at different infected time. As illustrated in Fig. 1A and B, the viral loads and virus titer were both gradually rising over the time at MOI=0.01, 0.1 and 1, suggesting that CV-A16 or CV-A10 could both reproduce in the SH-SY5Y cells. Moreover, the located expression of CV-A16 or CV-A10 in the SH-SY5Y cells was further confirmed by IF assay. The images presented CV-A16-VP1 protein (red) or CV-A10-VP1 protein (red) expressed in cytoplasm, which was around the nucleus (blue) (Fig. 1C). Therefore, our data revealed that CV-A16 or CV-A10 could both efficiently infected the SH-SY5Y cells.

### 3.2 CV-A16 and CV-A10 infections activate pyroptosis formation

**Fig. 2:**
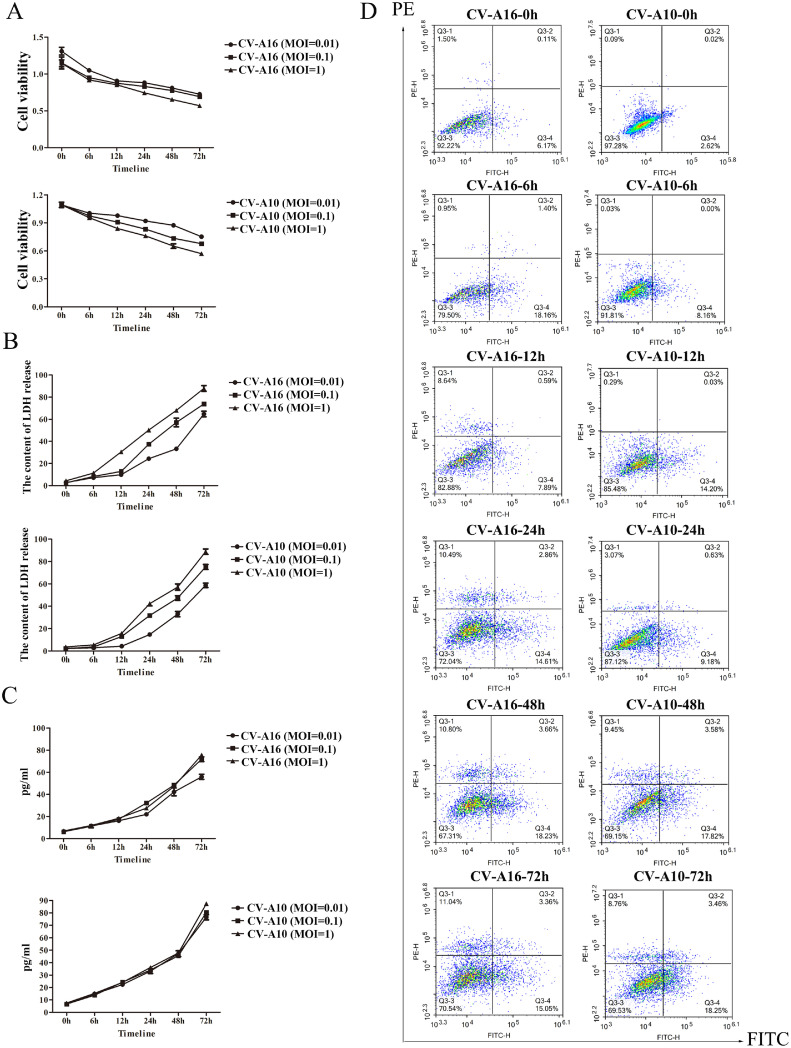
CV-A16 and CV-A10 induce the formation of pyroptosis. (A) Cell proliferation after inoculation with CV-A16 and CV-A10 of different MOIs was evaluated by CCK8 assay. (B) The intracellular LDH release after inoculation with CV-A16 and CV-A10 of different MOIs was measured with an LDH Cytotoxicity Assay Kit. (C) Caspase1 activity in supernatants from SH-SY5Y cells infected with CV-A16 or CV-A10 at different MOIs was examined via a Caspase1 Activity Assay Kit. (D) Cell death was detected by Annexin V-FITC/PI double staining using quantitative fluorescence-activated cell sorting (FACS) analysis after inoculation of CV-A16 or CV-A10 with MOI=0.1.

To systematically investigate whether CV-A16 and CV-A10 infections cause pyroptosis, cell viability was firstly examined by CCK8 assay. It was found that the cell viability was steadily downregulated over the time followed by CV-A16 or CV-A10 infection with MOI=0.01, 0.1 and 1 (Fig. 2A). Then, the LDH release analysis revealed that CV-A16 or CV-A10 infection promoted the cytotoxicity both dependent on MOI and time (Fig. 2B), which indicated that CV-A16 or CV-A10 infection might significantly disrupt cell membrane permeability. Next, caspase1 activity test also demonstrated that CV-A16 or CV-A10 infection resulted in a rising of caspase1 activity with the time (Fig. 2C). Pyroptosis is defined as Caspase-1-dependent programmed cell death, thereby our results might imply that CV-A16 or CV-A10 both tiggered pyrotosis. Next, flow cytometry experiment was further used to decide the types of cell death. As seen in Fig. 2D, the percentage of Q3-1 (representing late apoptotic cells or other dead cells), Q3-2 (representing mid-apoptotic cells) and Q3-4 (representing early apoptotic cells) were elevating all along with time after CV-A16 or CV-A10 infection, suggesting that CV-A16 or CV-A10 could induce other types cell death except for apoptosis, which might be speculated as pyroptosis.

**Fig. 3:**
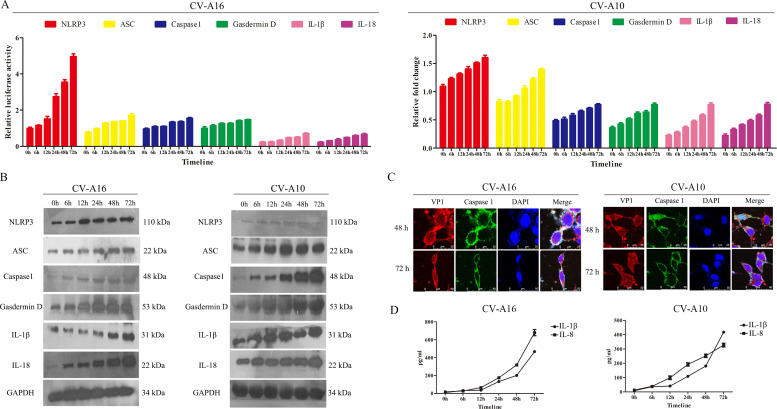
NLRP3-mediated pyroptosis of SH-SY5Y cells after CV-A16 and CV-A10 infection. (A) The expressions of NLRP3, ASC, Casepase1, Gasdermin D, IL-1β and IL-18 were measured by qRT-PCR in SH-SY5Y cells following CV-A16 and CV-A10 infections. (B) The levels of NLRP3, ASC, Casepase1, Gasdermin D, IL-1β and IL-18 were examined with WB in SH-SY5Y cells following CV-A16 and CV-A10 infections. (C) IF staining was used to identify CV-A16 or CV-A10-VP1 (red) and Caspase1 (green) in SH-SY5Y cells following CV-A16 and CV-A10 infections. (D) ELISA was applied to monitor the concentration of IL-1β and IL-18 in SH-SY5Y cells following CV-A16 and CV-A10 infections.

Afterwards, these molecules on the canonical pathway of pyroptosis were detected by qRT-PCR and WB assays. It was displayed that CV-A16 and CV-A10 promoted NLRP3, ASC, Caspase1, Gasdermin D, IL-1β and IL-18 expressions in mRNA and protein levels (Fig. 3A and B). Moreover, the CV-A16-VP1 or CV-A10-VP1 and caspase1 were both co-expressed in cytoplasm (Fig. 3C) performed by IF experiment, implying that CV-A16 or CV-A10 infection might activate caspase1. Additionally, it was also noticed that CV-A16 or CV-A10 infection improved IL-1β and IL-18 secretion (Fig. 3D). Thus, the above data disclosed that CV-A16 and CV-A10 infections might activate pyroptosis formation.

### 3.3 CV-A16 and CV-A10 infections contribute to elevated inflammatory response

**Fig. 4:**
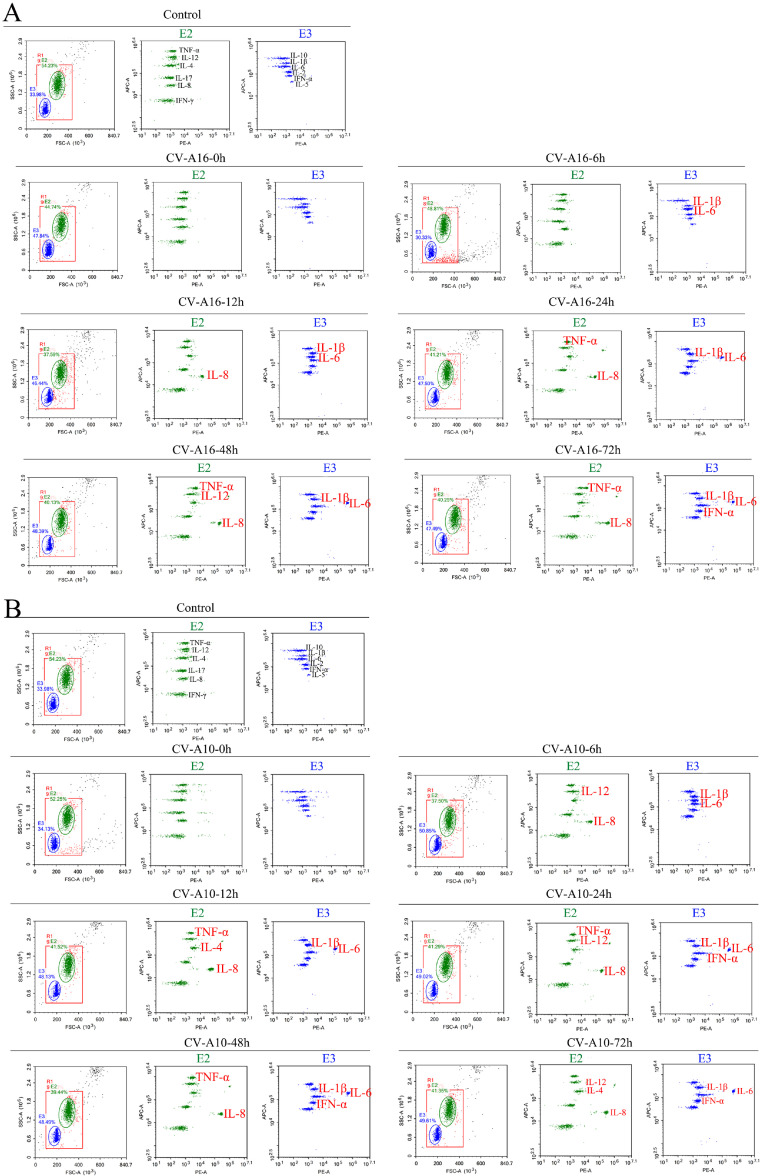
Impact of CV-A16 and CV-A10 on the inflammatory cytokines in SH-SY5Y cells. Concentrations of TNF-α, IL-12, IL-4, IL-17, IL-8, IFN-γ, IL-10, IL-1β, IL-6, IL-2, IFN-α and IL-5 in the culture media of CV-A16- and CV-A10-infected SH-SY5Y cells were determined by flow cytometry.

When infected with virus, the host recruits inflammatory cells to safeguard itself, which is largely mediated by inflammatory chemokines and cytokines (Casanova and Abel, 2021). However, excessive inflammatory response leads to severe inflammatory damage. Furthermore, pyroptosis is the process of inflammatory cell death, which could induce strong inflammatory responses. In the current study, flow cytometry was utilized to assess 12 inflammatory cytokines. As exhibited in Fig. 4, there were some inflammatory cytokines distinctly changed, especially TNF-α, IL-8, IL-1β, IL-6 and IFN-α. And the Table 2 clearly listed the concentrations of 12 inflammatory cytokines. It was discovered that TNF-α, IL-8, as well as IL-1β and IL-6, began to be upregulated persistently at 24 hpi, 12 hpi, and 6 hpi, respectively, during CV-A16 infection, and IL-12 and IFN-α were increased slightly in the later stages of CV-A16 infection. Additionally, it was also found that TNF-α and IFN-α began to be upregulated at 12 hpi and 24 hpi, respectively, during CV-A10 infection, but IL-8, IL-1β and IL-6 began to be upregulated at 6 hpi. Hence, these results pointed out that CV-A16 and CV-A10 infections might contribute to elevated inflammatory response.

**Table 2:** The changes of cytokine levels in CV-A16- and CV-A10-infected SH-SY5Y cells at different times.

Groups	TNF-α	IL-12	IL-4	IL-17	IL-8	IFN-γ	IL-10	IL-1β	IL-6	IL-2	IFN-α	IL-5
CV-A16-0h	1.2	0.51	0.46	0.62	0.53	1.09	0.41	0	0.69	0.8	2.57	0
CV-A16-6h	1.1	0.5	0.38	0.63	12.23	0.02	0.29	**38.13**	**6.66**	1.29	3.23	0
CV-A16-12h	10.2	0.77	6.43	2.4	**328.94**	0	2.51	**241.8**	**11.16**	2.59	5.71	0
CV-A16-24h	**23.78**	1.15	7.53	2.43	**2891.2**	1.56	2.59	**270.82**	**2978.62**	5.05	7.32	0
CV-A16-48h	**91.1**	**4.53**	8.33	3.18	**3224.72**	2.05	2.82	**296.25**	**4259.96**	5.83	8.43	0
CV-A16-72h	**152.24**	**4.04**	7.81	3.78	**3722.45**	3.07	2.8	**318.64**	**4759.06**	5.77	**12.58**	0
CV-A10-0h	1.22	0.54	0.59	0.64	3.98	1.33	0.44	9.14	1.57	0.87	1.47	0.48
CV-A10-6h	11.25	**4.09**	7.13	2.74	**542.68**	0.51	2.76	**296.01**	**23.7**	3.57	6.83	0
CV-A10-12h	**20.11**	2.92	**8.67**	2.16	**762.9**	0	2.58	**256.97**	**982.51**	4.71	6.69	0
CV-A10-24h	**34.99**	**3.43**	8.39	2.23	**3296.06**	0.12	2.68	**290.47**	**3522.55**	6.28	**9.22**	0
CV-A10-48h	**40.29**	**3.34**	7.71	2.31	**3554.8**	0	2.73	**311.21**	**4832.79**	6.33	**7.78**	0
CV-A10-72h	12.67	**3.47**	**9.27**	2.55	**4217.67**	0.04	2.79	**347.36**	**5495.79**	7.36	**10.17**	0

### 3.4 NLRP3 is involved in CV-A16- and CV-A10-induced pyroptosis

**Fig. 5:**
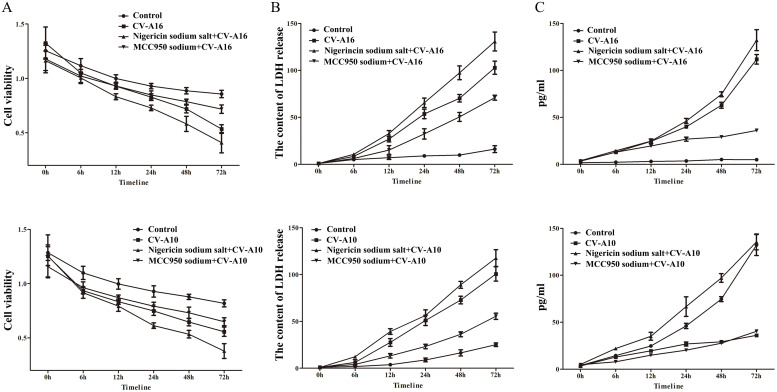
NLRP3 facilitates the cytotoxic effect during CV-A16 and CV-A10 infections. (A) Cell viability was evaluated from SH-SY5Y cells treated with CV-A16 and CV-A10-infection in the presence of Nigericin sodium salt or MCC950 sodium. (B) LDH release was measured from SH-SY5Y cells treated with CV-A16 and CV-A10-infection in the presence of Nigericin sodium salt or MCC950 sodium. (C) Caspase1 activity was tested from SH-SY5Y cells treated with CV-A16 and CV-A10-infection in the presence of Nigericin sodium salt or MCC950 sodium.

**Fig. 6:**
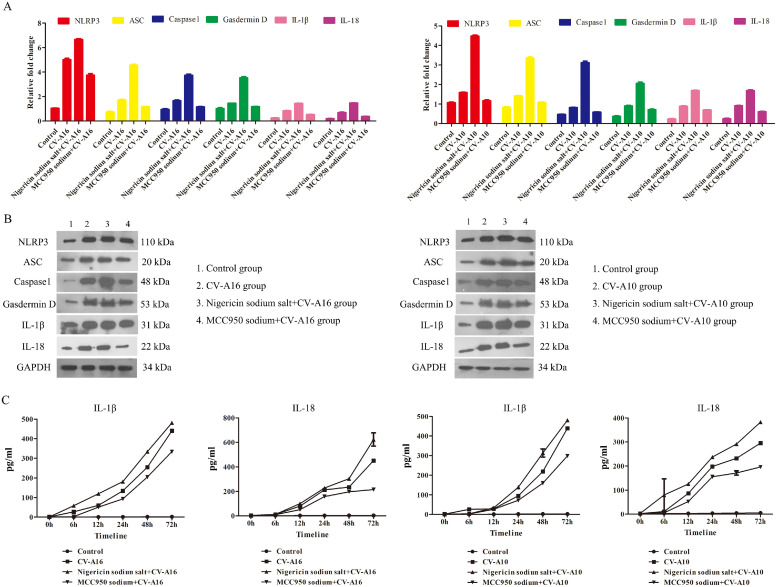
NLRP3 is responsible for pyroptosis induced by CV-A16 and CV-A10. (A) NLRP3, ASC, Casepase1, Gasdermin D, IL-1β and IL-18 mRNA expression levels in CV-A16 or CV-A10 infected SH-SY5Y cells pre-treated with Nigericin sodium salt or MCC950 sodium were analyzed by qRT-PCR. (B) WB assay of NLRP3, ASC, Casepase1, Gasdermin D, IL-1β and IL-18 from CV-A16 or CV-A10 infected SH-SY5Y cells pre-treated with Nigericin sodium salt or MCC950 sodium. (C) ELISA analysis of IL-1β and IL-18 in CV-A16 or CV-A10 infected SH-SY5Y cells pre-treated with Nigericin sodium salt or MCC950 sodium.

Emerging evidence has been reported that NLRP3 plays an important role in pyroptosis (Coll et al., 2022). Initially, NLRP3 was activated by pathogen-associated molecular patterns (PAMPs) and damage associated molecular patterns (DAMPs), such as viral RNAs, microbial toxins and bacterial surface components, uric acid crystals, aluminum adjuvant, and β-amyloid peptide; Then it recruits ASC and caspase1 to form the NLRP3-ASC-caspase1 protein complex, which is known as the NLRP3 inflammasome; The assembled NLRP3 inflammasome ultimately triggers the subsequent pyroptosis formation (Shi et al., 2017). To address whether NLRP3 was associated with CV-A16- and CV-A10-induced pyroptosis, the agonist and inhibitor of NLRP3 were used before CV-A16 and CV-A10 infections. Firstly, Nigericin sodium salt markedly reduced cell viability and elevated the LDH release and caspase1 activity upon CV-A16 or CV-A10 infection, while MCC950 sodium remarkably increased cell viability and decreased the LDH release and caspase1 activity upon CV-A16 or CV-A10 infection (Fig. 5). Next, the mRNA and protein levels of NLRP3, ASC, Caspase1, Gasdermin D, IL-1β and IL-18 were significantly ascended in SH-SY5Y cells stimulated with Nigericin sodium salt prior to CV-A16 or CV-A10 infection, but they were obviously descended in SH-SY5Y cells stimulated with MCC950 sodium prior to CV-A16 or CV-A10 infection (Fig. 6A and B). Furthermore, higher concentrations of IL-1β and IL-18 were observed in Nigericin sodium salt+CV-A16 group or Nigericin sodium salt+CV-A10 group, and lower concentrations of IL-1β and IL-18 were discovered in MCC950 sodium+CV-A16 group or MCC950 sodium+CV-A10 group than those in CV-A16 group or CV-A10 group, respectively (Fig. 6C).

**Fig. 7:**
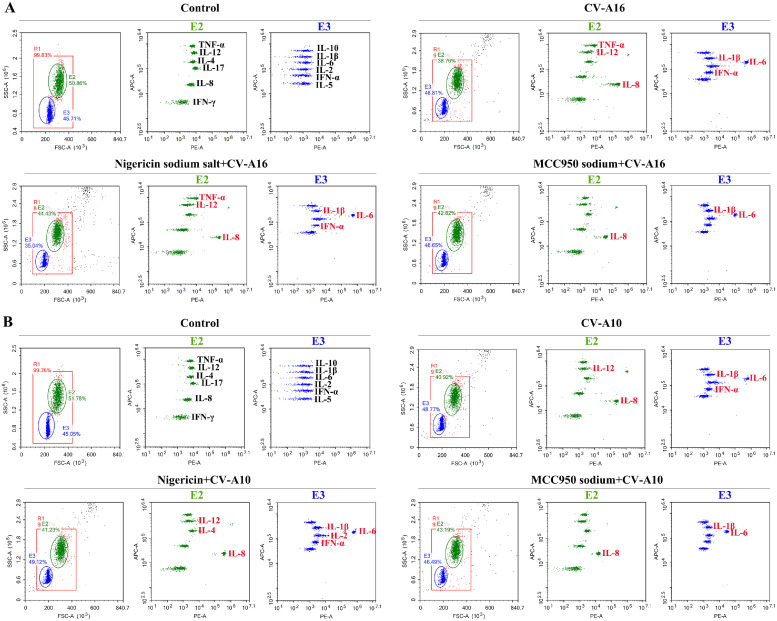
Effect of NLRP3 on inflammatory cytokines secretion in CV-A16 and CV-A10 infected SH-SY5Y cells. Flow cytometry of TNF-α, IL-12, IL-4, IL-17, IL-8, IFN-γ, IL-10, IL-1β, IL-6, IL-2, IFN-α and IL-5 in supernatants of SH-SY5Y cells stimulated by CV-A16 and CV-A10 in the presence of Nigericin sodium salt or MCC950 sodium.

Finally, to determine whether NLRP3 could influence the secretion of inflammatory cytokines in SH-SY5Y cells, flow cytometry assay was conducted. The results revealed that in contrast to CV-A16 infection, Nigericin sodium salt promoted inflammatory cytokines production (i.e., IL-8, IL-1β, IL-6 and IFN-α) pre-treated with CV-A16 infection, but MCC950 sodium attenuated the increase of inflammatory cytokines production (i.e., IL-8, IL-1β, IL-6 and IFN-α) with CV-A16 infection (Fig. 7A and Table 3). Meanwhile, it was also confirmed that Nigericin sodium salt resulted in enhanced inflammatory cytokines production (i.e., TNF-α, IL-8, IL-1β and IL-6) with CV-A10 infection, but MCC950 sodium resulted in attenuated inflammatory cytokines production (i.e., TNF-α, IL-8, IL-1β and IL-6) with CV-A10 infection (Fig. 7B and Table 3). Altogether, the above findings uncovered that NLRP3 might be involved in CV-A16- and CV-A10-induced pyroptosis.

**Table 3:** Effect of the NLRP3 on cytokines levels in CV-A16- and CV-A10-infected SH-SY5Y cells.

Groups	TNF-α	IL-12	IL-4	IL-17	IL-8	IFN-γ	IL-10	IL-1β	IL-6	IL-2	IFN-α	IL-5
Control	<1.21	<1.21	<1.1	<3.39	<2.26	1.06	5.9	<1.04	3.33	<4.38	<1.09	<2.79
CV-A16	**162.07**	**5.09**	8.11	4.48	**3253.12**	6.15	2.61	**293.41**	**4665.47**	5.91	**13.19**	0
Nigericin sodium salt+CV-A16	**211.36**	**4.07**	8.05	2.96	**3770.72**	3.07	2.64	**347.15**	**5026.49**	5.14	**12.52**	0
MCC950 sodium+CV-A16	16.19	1.04	6.55	2.17	**568.6**	0	2.53	**219.48**	**619.55**	3.44	4.71	0
Control	<1.25	<1.49	1.6	<3.1	<3.07	4.34	2.64	6.23	1.17	<7.92	4.4	<2.85
CV-A10	11.57	**3.54**	7.99	2.62	**3482.12**	0.43	2.55	**301.46**	**5712.97**	6.19	**10.77**	0
Nigericin sodium salt+CV-A10	12.67	**3.42**	**9.11**	2.37	**5393.74**	0.59	2.91	**397.7**	**7820.07**	**9.55**	**17.71**	0
MCC950 sodium+CV-A10	12.11	0.8	6.08	2.13	**242.65**	0	2.4	**166.21**	**228.74**	2.5	2.19	0

### 3.5 NLRP3 drives CV-A16 and CV-A10 infections

**Fig. 8:**
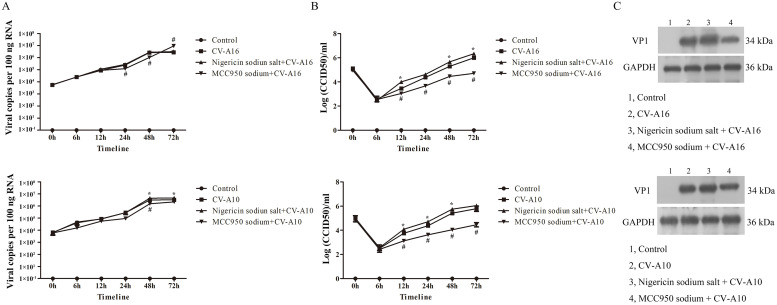
NLRP3 is critical for CV-A16 and CV-A10 replication. (A) A qRT-PCR with TaqMan analysis was used to examine the viral copies of CV-A16. (B) A plaque assay was applied to identify the virus titer of CV-A16 and CV-A10. (C) A WB method was utilized to determine the VP1 protein expression level of CV-A16 and CV-A10. Compared with the infection groups, the groups pretreated with Nigericin sodium salt was marked with *, while the groups pretreated with MCC950 sodium was marked with #.

To discuss whether NLRP3 could influence CV-A16 and CV-A10 propagation, the viral loads, virus titer and VP1 protein expression were further detected. In compared to CV-A16 or CV-A10 infection, the viral loads, virus titer and VP1 protein expression were all distinctly up-regulated in SH-SY5Y cells with Nigericin sodium salt pre-treatment, while they were all notably down-regulated in SH-SY5Y cells with MCC950 sodium pre-treatment (Fig. 8). Consequently, these results demonstrated that NLRP3 might drive CV-A16 and CV-A10 infection.

## 4 Discussion

Historically, EV-A71 and CV-A16 have been the dominant pathogens for HFMD (Aswathyraj et al., 2016) However, with the arrival of the EV-A71 inactivated vaccine, the pathogen spectrum of HFMD has dramatically altered (Jiang et al., 2021; Liu et al., 2020). In recent years, it was found that CV-A6 and CV-A10 have replaced EV-A71 and CV-A16 as the predominant serotypes for HFMD prevalence in mainland China (Chen et al., 2014; Wang et al., 2021). Normally, the infection of most enteroviruses is sub-clinical and self-resolving that only lasts a few days without complications, but a small proportion of patients develops severe neurologic and cardiopulmonary complications like meningitis, encephalitis, acute respiratory tract infections, viral myocarditis, and even fatalities (Tikute and Lavania, 2023; Zhu et al., 2023). Previous studies, whether at the cellular level, animal models or clinical samples, have suggested that enteroviruses have a strong neurotropism, and the CNS damages caused by enteroviruses might be the premise of CNS complications and even death of patients (Tikute et al., 2019; Wang et al., 2003, 2004b). However, to date, it remains unclear about the underlying neuropathological mechanism of enteroviruses. Moreover, it is well known that although the vaccination of EV-A71 inactivated vaccine has greatly reduced the infection of EV-A71 in children, the vaccine has no protective effect on other subtypes of enteroviruses, including CV-A16 and CV-A10 (Bian et al., 2019; Mao et al., 2014). Therefore, this work aimed to explore the neuropathogenesis of CV-A16 and CV-A10, which have high infection rates at present, and searched for their commonalities, which may provide new directions and ideas for their diagnosis and treatment. Several lines of studies have reported that cell death and inflammatory damage in the central nervous system are key factors that trigger severe and critical illness in many neurotropic viruses, including enteroviruses (Furr and Marriott, 2012; Wong et al., 2008). Pyroptosis, a novel inflammatory form of programmed cell death, have been recognized to exhibit a significant role host defense by promoting the formation of inflammatory responses and triggering innate immune responses against viruses, but excessive inflammatory responses can lead to severe immunopathology and fatal outcomes during viral infection (Bergsbaken et al., 2009; Kesavardhana et al., 2020). So, pyroptosis actually is a double‐edged sword during the process of virus infection (Liu et al., 2023). Currently, accumulating evidence has verified that the execution of pyroptosis depends on the formation of large cytosolic protein complexes termed inflammasomes, and large amount of viruses could drive pyroptotic cell death via activating different inflammasomes (Rao et al., 2022; Yu et al., 2021). For example, Hepatitis B virus X protein promotes liver cell pyroptosis under oxidative stress through NLRP3 inflammasome activation (Xie et al., 2020). SARS-CoV-2 infects blood monocytes to activate NLRP3 and AIM2 inflammasomes, pyroptosis and cytokine release (Junqueira et al., 2021). Zika virus is able to stimulate AIM2 expression and the secretion of IL-1β in primary human skin fibroblasts, eventually resulting in an elevated inflammatory response (de Sousa et al., 2018). However, among those inflammasomes, NLRP3 is one of the most well-studied inflammasome in canonical pyroptotic pathway (Coll et al., 2022). Thus, in this study, we primarily focused on the mechanism investigation about the role of NLRP3-dependent pyroptosis in inflammatory response and viral replication during CV-A16 and CV-A10 infections. It was found that CV-A16 and CV-A10 infections both induced pyroptotic cell death in MOI- and time-dependent manners in SH-SY5Y cells. Additionally, it was further uncovered that the NLRP3 inflammasome was activated, and NLRP3-mediated downstream pyroptotic pathway was also promoted, finally leading to elevating IL-1β and IL-18 secretion, and meanwhile the inflammatory cytokines were significantly increased. Then, in order to further explore the role of NLRP3, the agonist and inhibitor of NLRP3 were applied for the following experiments. Our data precisely displayed that Nigericin sodium salt treatment reduced the cell viability, enhanced the release of LDH and elevated the Caspase1 activity in CV-A16- and CV-A10-infected cells, but MCC950 treatment caused opposite results. Furthermore, Nigericin sodium salt treatment also significantly improved the NLRP3-mediated pyroptosis pathway, resulting in an overwhelming release of IL-1β and IL-18, while MCC950 treatment partially suppressed the above effects. Meanwhile, Nigericin sodium salt treatment further aggravated the release of inflammatory cytokines caused by CV-A16 or CV-A10 infection, whereas MCC950 treatment weakened the release of inflammatory cytokines caused by CV-A16 or CV-A10 infection. Taken together, these results probably speculated that NLRP3-dependent pyroptosis might contribute to CV-A16 and CV-A10 pathogenesis, especially inflammatory pathological injury.

In addition to the role of NLRP3 in viral pathogenesis, it was continued to examine the effect of NLRP3 in viral replication in this work. Several studies have reported that cell death provide an effective defense against pathogen infection by limiting replication of the invasive viruses within cells; however, on the other hand, viruses have more complex immune evasion mechanisms to favor viral replication using the cell death pathways (Imre, 2020; Stephenson et al., 2016). For instance, dual inhibition of innate immunity and apoptosis by human cytomegalovirus protein UL37 × 1 enables efficient virus replication, but Caprine parainfluenza virus type 3N protein promotes viral replication via inducing apoptosis (Ren et al., 2022). RIPK3-dependent necroptosis is induced and restricts viral replication in human astrocytes infected with Zika Virus (Wen et al., 2021), while Rotavirus activates MLKL-mediated host cellular necroptosis concomitantly with apoptosis to facilitate dissemination of viral progeny (Mukhopadhyay et al., 2022). Additionally, it was reported that AIM2 and NLRP3 inflammasome-mediated pyroptosis in EV-A71-infected neuronal cells restricts viral replication (Hu et al., 2023; Yogarajah et al., 2017). In the current study, we also paid our attention on the effect of NLRP3 inflammator-mediated pyroptosis on viral replication, and discovered that the activated NLRP3 facilitated the CV-A16 and CV-A10 replication in infected SH-SY5Y cells, and vice versa, probably pointing out that NLRP3 promote a productive infection of CV-A16 and CV-A10. In fact, many studies have revealed that NLRP3 not only promoted virus replication, but also limited virus replication (Shrivastava et al., 2016). For instance, NLRP3 inflammasome was involved with viral replication of bovine viral diarrhea virus (Gallegos-Rodarte et al., 2023). Knockdown of NLRP3 benefited newcastle disease virus replication in the host cells (Wang et al., 2016). Thus, why NLRP3 has a double-sided effect on virus infection mainly depends on the game between virus and host immunity.

## 5 Conclusions

In summary, this work provides the first demonstration that CV-A16 and CV-A10 can induce inflammatory responses via triggering NLRP3 inflammasome activation and pyroptosis in SH-SY5Y cells, suggesting that NLRP3-dependent pyroptosis might be an important contributing factor for inducing exacerbated inflammation during CV-A16 and CV-A10 infections. Moreover, it was further revealed that NLRP3 also enhanced the virus replication of CV-A16 and CV-A10. Hence, NLRP3-mediated pyroptosis might represent a novel anti-inflammation strategies and treatments for the prevention of severe immunopathology induced by CV-A16 and CV-A10.

## CRediT authorship contribution statement

**Yajie Hu:** Conceptualization, Writing – original draft, Funding acquisition. **Wei Zhao:** Software, Investigation. **Yaming Lv:** Methodology, Software. **Hui Li:** Data curation, Methodology. **Jiang Li:** Software, Data curation. **Mingmei Zhong:** Software, Data curation. **Dandan Pu:** Methodology. **Fuping Jian:** Visualization, Investigation. **Jie Song:** Conceptualization, Writing – review & editing, Funding acquisition. **Yunhui Zhang:** Conceptualization, Supervision, Writing – review & editing.

## Declaration of competing interest

The authors declare that the research was conducted in the absence of any commercial or financial relationships that could be construed as a potential conflict of interest.

## Data Availability

Data will be made available on request.
